# Oral Health Profiles and Related Quality of Life in Thalassemia Children in Relation to Iron Overload: A Cross-Sectional Study

**DOI:** 10.3390/ijerph17249444

**Published:** 2020-12-16

**Authors:** Hani T. Fadel, Mohammed A. Zolaly, Manal O. Alharbi, Lujain A. Qarah, Maher S. Alrehili, Abdulaziz D. Alamri, Ahmed M. Tarawah

**Affiliations:** 1 Department of Preventive Dental Sciences, College of Dentistry, Taibah University, AlMadinah AlMunawwarah 42313, Saudi Arabia; 2 Department of Pediatrics, College of Medicine, Taibah University, AlMadinah AlMunawwarah 42353, Saudi Arabia; mazolaly@hotmail.com; 3 Private Practice, AlMadinah AlMunawwarah 42351, Saudi Arabia; dentistmanala@gmail.com (M.O.A.); lojainqa@gmail.com (L.A.Q.); maheralrehili@gmail.com (M.S.A.); azozalbadrani@gmail.com (A.D.A.); 4 Pediatric Hematology, King Abdullah Medical City, AlMadinah AlMunawwarah 42319, Saudi Arabia; tarawah@yahoo.com

**Keywords:** beta-thalassemia, iron overload, oral health, quality of life

## Abstract

The aim was to assess the oral health of children with β-thalassemia major (BTM) and their oral health-related quality of life (OHRQoL) in relation to the serum ferritin level (SFL). Thirty-nine children with BTM underwent an interview, salivary sampling and an oral clinical examination. The Early Childhood Oral Health Impact Scale (ECOHIS) was used to assess their OHRQoL. The mean age of the participants was 9 ± 3 years, with 62% females. The body mass index and salivary secretion rate were within normal ranges. The mean plaque index, gingival bleeding index and number of decayed, missing and filled tooth surfaces were 70 ± 29, 38 ± 25 and 3.2 ± 4, respectively, with no significant differences between individuals with SFL below or above 2000 ng/mL (*p* > 0.05). No significant differences were observed between the two groups in any of the ECOHIS questions (*p* > 0.05). The mean ECOHIS score was 4.2 ± 4. Individuals with SFL ≥2000 ng/mL had a significantly higher mean score in the family domain “Parent Distress” than those with lower SFL (*p* ≤ 0.05). Within the study limits, children with β-thalassemia major generally had high dental caries experience and gingival inflammation, yet an acceptable OHRQoL. Those with high SFL had less favorable scores in the domain “Parent Distress”.

## 1. Introduction

Thalassemia is a chronic autosomal recessive disorder that is characterized by severe anemia and requires blood transfusion for life [1]. As a result, patients with this condition are susceptible to the development of transfusion-related toxicity due to iron overload [2]. Iron chelation therapy is necessary to avoid lifelong complications that may arise as a result of iron overload [3]. The two main sources of iron overload are continuous blood transfusion and increased intestinal iron absorption [3]. The serum ferritin level (SFL) is used worldwide as a measure of chelation efficacy. Many centers consider an SFL between 500 ng/mL and 1500 ng/mL as an acceptable iron control level [4]. An SFL of more than 2500 ng/mL may reliably predict cardiac and endocrine disease [3].

Thalassemia directly impacts the physical and psychological wellbeing of affected individuals and their families [5]. Effects have serious implications on the physical, emotional, social, and school functioning of patients, consequently leading to an impaired quality of life [6]. Contributory factors that influence the health-related quality of life in thalassemia patients include continuous hospital admissions, the need for chelation therapy and uncertainties about the future [7,8]. As the development of iron overload occurs gradually, the impact on the quality of life in thalassemia patients will not be visible until major complications such as cardiovascular disease and diabetes have occurred [9,10].

Oral conditions such as dental caries and periodontal disease are amongst the most common infections to affect man. Oral and maxillofacial features and complications have been reported in patients with thalassemia [11]. Facial symptoms of these patients result from extra-medullary hematopoiesis and compensatory growth of the bone marrow [12]. The observed maxillary enlargement can cause structural changes in the oral cavity such as teeth protrusion, spacing, occlusal deep bite, open bite and different degrees of malocclusion that predispose to dental problems [13]. High degrees of periodontal diseases and dental caries have been extensively reported in thalassemia patients [14,15,16]. In addition, patients may suffer from other oral conditions due to neglect of oral hygiene, increased level of serum ferritin, iron overload and the variations in salivary biochemical constituents [17]. The variations in salivary composition may be due to iron overload, which can result in hypogonadism, diabetes and other endocrine abnormalities [15].

The child’s oral health has an impact on eating, talking, laughing and appearance. Pain that results from oral health problems may negatively affect the child’s daily life [18]. The oral health-related quality of life (OHRQoL) is a multidimensional construct which is mainly used to assess the individual’s own perceptions and feeling of the impact of an oral disease or condition [19]. Amirabadi et al. found a significant relationship between dental caries and OHRQoL in children with β-thalassemia major (BTM) [20]. Furthermore, the OHRQoL declined as oral health problems increased. A recent study also reported a negative impact of thalassemia on the OHRQoL [21].

To the best of our knowledge, no studies have been published that looked into the relation between iron overload and the oral health-related quality of life in individuals with thalassemia. The aims of this study were thus to assess the oral health of children with β-thalassemia major, and to evaluate their oral health-related quality of life in relation to iron overload.

## 2. Materials and Methods

### 2.1. Study Design and Sample

This was a hospital-based, cross-sectional analytic investigation. The study sample comprised children diagnosed with BTM, who were attending the Madinah Hereditary Blood Disorders Center at the Maternity and Children Hospital in AlMadinah AlMunawwarah, Saudi Arabia for their follow up and blood transfusion sessions. The hospital is one of three major governmental hospitals in the region that are run by the Ministry of Health, and is considered a tertiary referral center for the west part of the country, with a capacity of 500 beds, 300 of which are for pediatric patients. Each year, the center receives around 1200 patients with different hematologic illnesses and needs, accounting for up to 4000 treatment or follow up visits per year.

Given the rare nature of the disease and the limited number of attending patients, the total population of BTM patients who were attending at the center were included in the study. All included participants sat for an interview with their guardian(s), had their saliva sampled and underwent a clinical oral examination.

### 2.2. Interview and Questionnaire

The guardian(s) were first asked to provide information related to their child’s demographic, lifestyle and general health aspects. Other necessary personal and health-related data were collected from the patient’s medical record on a customized information sheet. These included the patient’s age, height, weight, dietary habits and personal hygiene. Collected systemic health information included concurrent medical conditions, last record of serum ferritin level and the hemoglobin level.

Guardians and their children (>6 years old) answered a validated Arabic version [22] of the Early Childhood Oral Health Impact Scale (ECOHIS-13) [23], which has proven to be an acceptable tool for assessing OHRQoL in young children [24]. Children who were 6 years old or younger had their guardians answer in full. The scale is a structured, self-administered, closed-ended 5-point Likert scale questionnaire that includes 13 questions under two sections; Child Impact Oral Health (CIOH) and Family Impact Oral Health (FIOH). The CIOH consists of four domains and nine items, with a possible score range from 0 to 36. These domains are symptoms (1 question), function (4 questions), psychology (2 questions), and self-image and social interaction (2 questions). The FIOH, on the other hand, has two domains and four items, with the possible score ranging between 0 and 16. The domains are parental distress (2 questions) and family function (2 questions). Accordingly, lower ECOHIS-13 scores reflect better oral health related quality of life (OHRQoL), while higher scores reflect greater impact, more oral health problems, and poor OHRQoL [23].

### 2.3. Saliva Sampling

Saliva samples were collected from all participants according to the method described by Heintze et al. to determine the resting salivary flow rate [25]. Briefly, participants were instructed to wash their mouth with distilled water before sampling. Unstimulated saliva was collected while the participant sat in an upright position and slightly inclined to the front with the head faced down and mouth opened, allowing drooled saliva to be collected in a glass vial for 5 min. All samples were collected in the morning.

### 2.4. Oral Clinical Examination

An intraoral clinical examination was performed on all participants in an isolated room at the Hematology and Oncology Center, utilizing a portable dental chair, an optimal light source, a graded Williams periodontal probe (Hu-Friedy, Chicago, IL, USA), a mouth mirror and sterile cotton rolls. Four dental graduates, who were trained and calibrated by an experienced periodontist; performed the examinations. The number of decayed, missing and filled tooth surfaces was determined from all teeth. The plaque control record (PCR) [26] and the gingival index (GI) [27] were registered at four tooth sites; mesial, distal, buccal and lingual, from two crossed upper and lower quadrants, selected by means of a coin flip.

### 2.5. Ethical Considerations

The protocol was approved by the Taibah University College of Dentistry Research Ethics Committee (approval no. TUCDREC/20180107/Qarah) as well as the Institutional Review Board at the General Directorate of Health Affairs in Madinah (approval no. 120–07/02/2018). It respected the ethical principles outlined in the Declaration of Helsinki [28], and the guidelines for strengthening the reporting observational studies in epidemiology (STROBE) were also followed [29]. Prior to commencement of the study, all participants and their guardian(s) were informed about the study objectives and the procedures involved. They were assured that any gathered information was to remain confidential, and would only be used for research and educational purposes. They were also informed that participation was voluntary, and that no negative impact on the provided medical services would result if they chose not to participate. Upon approval, the guardian(s) signed a written informed consent form on their child’s behalf. Participants with apparent oral health problems were offered free treatment at the Taibah University Dental College and Hospital clinics.

### 2.6. Data Analysis

Descriptive statistics in the form of means and standard deviations for continuous variables and frequencies and percentages for categorical variables, were used. The Mann–Whitney U and chi-square tests were used to compare between groups with SFL below and above 2000 ng/mL. The significance level was set at 0.05. The statistical software IBM^®^ SPSS^®^ (version 20) was used for the analysis (IBM, Armonk, NY, USA).

## 3. Results

In total, 39 follow up individuals with BTM participated in this study, 62% of which were females (Table 1). None of the center attendees were undergoing dental treatment nor received dental prophylaxis during the six months that preceded the study. The mean age of the participants was 9 ± 3 years, with 9 participants being 6 years old or below (Table 1). The body mass index (15.3 ± 2 kg/m^2^) and the salivary secretion (2.8 ± 1 mL/min) were within normal range (Table 1). Seventy-two percent of the participants had undergone splenectomy, and the mean SFL was 2733 ± 2919 ng/mL. Those with SFL < 2000 ng/mL accounted for 51% of the sample (n = 20).

Scores for dental plaque and gingival bleeding were high in individuals with SFL below or above 2000 ng/mL (*p* > 0.05). Similarly, the mean number of decayed, missing and/or filled tooth surfaces was 3.2 ± 4 in the studied sample, with no significant differences observed between the two groups (*p* > 0.05) (Table 1).

**Figure 1:**
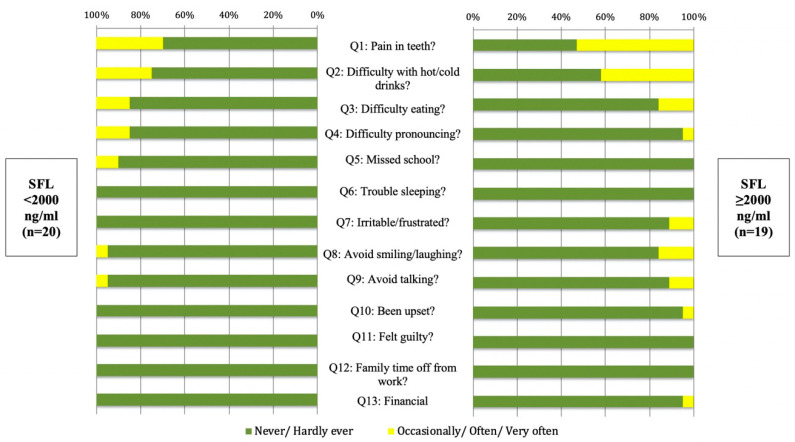
Stacked bar-chart showing the response percentages of the β-thalassemia major (BTM) participants with serum ferritin level (SFL) <2000 ng/mL (n = 20) and with SFL ≥2000 ng/mL (n = 19) to all 13 questions of the Early Childhood Oral Health Impact Scale (ECOHIS). No significant differences between the groups were detected using the chi-square test (*p* > 0.05).

Table 2 shows the responses from all participants to the 13 questions of the ECOHIS, while Figure 1 demonstrates the responses of the participants from each group to those questions. Fifty-three percent of the individuals with SFL ≥2000 ng/mL occasionally or often experienced dental pain, opposed to only 30% of those with SFL <2000 ng/mL (*p* > 0.05). Similarly, 42% of the SFL ≥2000 ng/mL group occasionally or often had difficulties consuming hot and/or cold beverages, compared to 25% of the individuals with SFL <2000 ng/mL (*p* > 0.05). Responses of both groups to the remaining questions were mostly ever or hardly ever experienced (range 84–100%). The remainder of the questions pertained to difficulties in eating, pronunciation, sleeping, socializing, the feel of frustration or guilt, missing school, and parental work or financial impact. Overall, no significant differences were observed between the two groups with regards to any of the 13 posed questions (*p* > 0.05).

**Figure 2:**
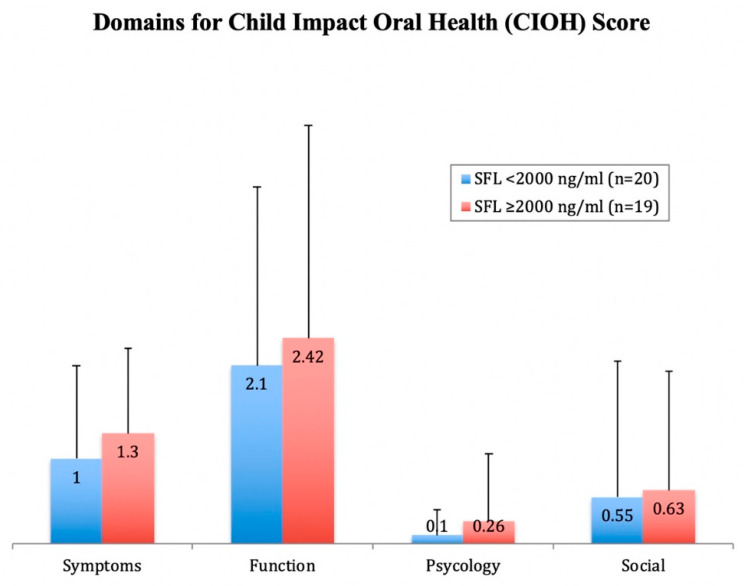
Bar-chart showing mean scores for the Child Impact Oral Health (CIOH) section domains in β-thalassemia major (BTM) participants with serum ferritin level (SFL) <2000 ng/mL (n = 20) and with SFL ≥2000 ng/mL (n = 19). No significant differences between the groups were detected using the Mann–Whitney U test (*p* > 0.05).

With regards to the CIOH section, the mean score was 4.2 ± 4, with no significant differences observed between the two groups (*p* > 0.05) (Table 1). Figure 2 shows the scores of the different domains within the CIOH section, with the highest related to the domain “Function”, followed by “Symptoms”. No significant differences between the two groups were observed (*p* > 0.05).

**Figure 3:**
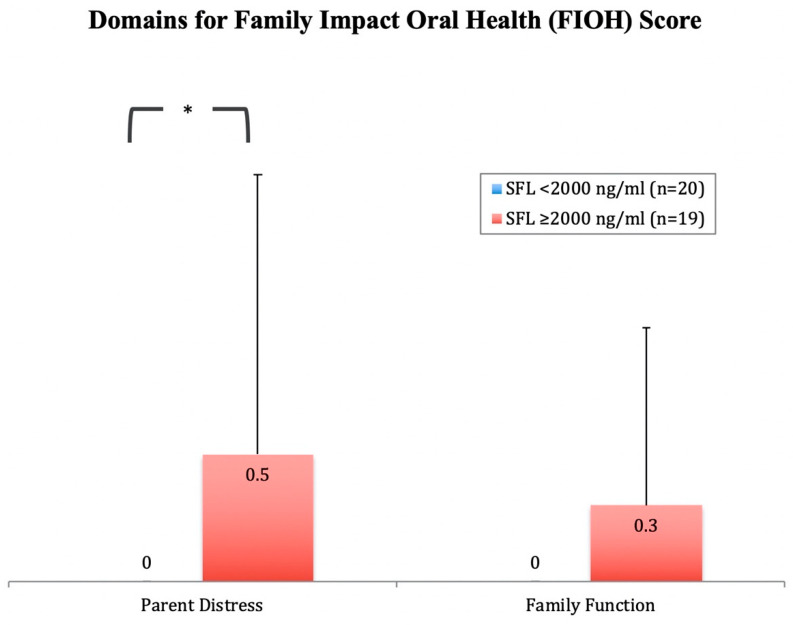
Bar-chart showing mean scores for the Family Impact Oral Health (FIOH) section domains in β-thalassemia major (BTM) participants with serum ferritin level (SFL) <2000 ng/mL (n = 20) and with SFL ≥2000 ng/mL (n = 19). A significant difference between the groups was detected in the domain “Parent Distress” using the Mann–Whitney U test (*p* ≤ 0.05). * Statistically significant at the 0.05 level using the Mann–Whitney U test.

Similarly, the mean score for the FIOH section was 0.4 ± 1. Individuals with SFL ≥ 2000 ng/mL had a significantly higher score for this section (*p* ≤ 0.05) (Table 1). Figure 3 shows the scores for the two domains within the FIOH section. Individuals with SFL ≥2000 ng/mL demonstrated a significantly higher score for the domain “Parent Distress” (*p* ≤ 0.05).

## 4. Discussion

This study aimed to assess the clinical oral health parameters and the oral health-related quality of life in relation to the serum ferritin level (SFL) of children with β-thalassemia major (BTM). The mean number of decayed, missing and filled tooth surfaces was 3.2. This was in line with many studies reporting that BTM patients are more prone to dental caries [15]. A recent study showed that thalassemia individuals had significantly higher caries than healthy controls [20]. This could be due to several predisposing factors including the difference in the tooth morphological features such as pits, fissure, tubercles, prominences and protuberances, in addition to the observed differences in salivary volume and constituents [15].

No significant differences were found between individuals with SFL below or above 2000 ng/mL with regard to salivary secretion rates and, consequently, dental caries. This was despite what is mentioned in the literature that the severity of dental caries increases due to the elevated ferritin levels in saliva and teeth, and that the quality of saliva is affected by elevated serum ferritin, which has a direct effect on the salivary glands and may contribute to dental caries [15]. Moreover, frequent blood transfusion in BTM patients has an impact on general as well as oral health, and there is a significant correlation between the effect of blood transfusion and dental caries [15]. The absence of significant differences in the current investigation could in part be due to the small sample size.

With regard to periodontal health, participants generally presented with moderately high levels of gingivitis, regardless of their SFL. Interestingly, a study in France reported that the severity of periodontitis in adults increased as the serum biomarkers of iron burden increased as well [30]. This may be attributed to the reversible nature of gingivitis, and the young age group of the current investigation, as irreversible conditions such as periodontitis have a low prevalence in younger populations and are known to appear more evidently at a later age [31]. Accordingly, a more notable association with iron overload can be assumed eventually.

The overall mean score for the Early Childhood Oral Health Impact Scale (ECOHIS) was low, indicating an acceptable oral health-related quality of life (OHRQoL). This follows a previous report highlighting the impact of improving patient care on the overall quality of life [32]. However, and although not significant, the ECOHIS score was somewhat higher in participants with high SFL than those with lower SFL, suggesting poorer OHRQoL. This follows what is mentioned in the literature that the impact on OHRQoL may only be evident later in life, given the gradual cumulative effect of iron overload and the delayed appearance of associated serious complications [6].

With regard to the child impact oral health (CIOH) section domains, pain, difficulty with hot or cold drinks, eating problems and issues with smiling were the most frequently reported in the studied sample. This corroborates well with the findings by Phrai-in and co-workers who reported issues with eating, personal oral hygiene maintenance and smiling to be the most common among thalassemia patients [14]. Such findings point to the devastating and widespread impact BTM may have on affected individuals, and calls for continuous psychological and therapeutic support.

Interestingly, patients with serum ferritin above 2000 ng/mL were higher in the family impact oral health (FIOH) score. Parent distress in particular was significantly higher than in those with lower serum ferritin. This finding can be understood since continuous visits to the hospital for blood transfusion are required for these patients. Families and guardians are consequently put under immense pressure to cope with the regular appointments, specific treatment needs and associated expenses [5,9]. Moreover, the regular functions of the family at home and/or work are affected, along with the psychological impact and implications of their child’s illness [15].

The current investigation involved a small sample, which may have an impact on the representativeness of the results. However, all patients following up at the selected tertiary care center were included, which gives a good picture on affected individuals in the region. In addition, and given the rare nature of β-thalassemia major, the literature is still in need of studies attempting to cover different aspect of the affected individuals, despite the acknowledged shortcomings in the study design.

## 5. Conclusions

Within the limits of this study, it can be concluded that children with β-thalassemia major had high dental caries experience and gingival inflammation. No significant differences were observed between children with high and low serum ferritin levels. β-Thalassemia children generally had an acceptable oral health-related quality of life (OHRQoL). However, those with high serum ferritin levels had less favorable OHRQoL scores in the family oral health impact domain “Parent Distress”.

This study suggests that parents of children with β-thalassemia major should be aware of the negative effect of iron overload on their children’s oral health-related quality of life and should be educated regarding the importance of maintaining good oral hygiene. Policymakers also need to take necessary measures to facilitate the frequent, lifelong visits of such patients and ways to reduce the stress on affected families psychologically, practically and financially.

## Figures and Tables

**Table 1:** Demographic variables, oral health parameters and section and overall scores for the Early Childhood Oral Health Impact Scale (ECOHIS) in all participants with β-thalassemia major (BTM) (n = 39), including those with serum ferritin level (SFL) <2000 ng/mL (n = 20) and with SFL ≥2000 ng/mL (n = 19).

Variable	All BTM Participants (n = 39)	Participants with SFL <2000 ng/mL (n = 20)	Participants with SFL ≥2000 ng/mL (n = 19)	*p*-Value
Age-years (±SD)	9 (3)	9 (3)	10 (3)	0.683
Gender-F/M (%)	24 (62)/15 (38)	11 (55)/9 (45)	13 (68)/6 (32)	0.389
Body Mass Index–kg/m^2^ (±SD)	15.3 (2)	15.6 (2)	15.1 (3)	0.593
Saliva Secretion rate-mL/min (±SD)	2.8 (1)	3.1 (1)	2.6 (1)	0.538
Plaque Index-% (±SD)	70 (29)	73 (31)	67 (28)	0.877
Gingival Bleeding Index-% (±SD)	38 (25)	40 (25)	37 (26)	0.944
Decayed, missing and filled tooth surfaces (±SD)	3.2 (4)	3.0 (4)	3.4 (3)	0.349
Number of decayed tooth surfaces (±SD)	2.3 (3)	1.9 (2)	2.8 (3)	0.399
Number of missing tooth surfaces (±SD)	1 (1)	0.4 (1)	0.6 (1)	0.280
Number of filled tooth surfaces (±SD)	0 (0)	0.1 (0)	0.0 (0)	0.330
Early Childhood Oral Health Impact Scale (ECOHIS) (±SD)	4.5 (4)	3.8 (4)	5.3 (5)	0.334
Child Impact Oral Health (CIOH) Section (±SD)	4.2 (4)	3.8 (4)	4.6 (4)	0.598
Family Impact Oral Health (FIOH) Section (±SD)	0.4 (1)	0.0 (0)	0.7 (2)	0.033 *

* Statistically significant at the 0.05 level using the Mann–Whitney U test.

**Table 2:** Frequency distribution (and percentages) for responses of all β-thalassemia major (BTM) participants (n = 39) to the 13 questions of the Early Childhood Oral Health Impact Scale (ECOHIS).

Domain	Never/Hardly Ever	Occasionally/Often/Very Often
**Question**	(%)	(%)
***Child Symptoms Domain***		
1. How often has your child had **pain** in the teeth, mouth or jaws?	23 (59)	16 (41)
***Child Function Domain***		
How often has your child … because of dental problems or dental treatments?		
2. had **difficulty drinking hot or cold beverages?**	26 (67)	13 (33)
3. had **difficulty eating some foods?**	33 (85)	6 (15)
4. had **difficulty pronouncing any words?**	35 (90)	4 (10)
5. **missed preschool, daycare or school?**	37 (95)	2 (5)
***Child Psychological Domain***		
How often has your child … because of dental problems or dental treatments?		
6. had **trouble sleeping?**	39 (100)	0 (0)
7. been **irritable or frustrated?**	37 (95)	2 (5)
***Child Self-Image/Social Interaction Domain***		
How often has your child … because of dental problems or dental treatments?		
8. **avoided smiling or laughing** when around other children?	35 (90)	4 (10)
9. **avoided talking** with other children?	36 (92)	3 (8)
***Parent Distress Domain***		
How often have you or another family member … because of your child’s dental problems or dental treatments?		
10. been **upset?**	38 (97)	1 (3)
11. felt **guilty?**	39 (100)	0 (0)
***Family Function Domain***		
How often …		
12. have you or another family member **taken time off from work** because of your child’s dental problems or dental treatments?	39 (100)	0 (0)
13. has your child had dental problems or dental treatments that had a **financial impact** on your family?	38 (97)	1 (3)
